# 8. DECREASING CARDIOVASCULAR RISK IN PERSONS WITH SCHIZOPHRENIA: INTERVENTIONS AND FUTURE DIRECTIONS

**DOI:** 10.1093/schbul/sby014.027

**Published:** 2018-04-01

**Authors:** Gail Daumit

**Affiliations:** Johns Hopkins Medical Institutions

## Abstract

**Overall Abstract:** Persons with schizophrenia experience two to three times higher mortality than the overall population. This premature death is due in large part to cardiovascular disease and is potentially preventable. All cardiovascular risk factors are elevated in persons with schizophrenia. This presentation will describe the evidence for interventions to reduce cardiovascular risk factors in this vulnerable population, including obesity and tobacco smoking, and will describe models of integrated physical and mental health care. Ongoing research on interventions to decrease cardiovascular risk in schizophrenia will be presented, and future research needs will be discussed including implementation strategies to scale-up interventions to reduce cardiovascular disease risk in community settings.

